# The impact of the COVID-19 pandemic on pediatric acute conjunctivitis disease trends

**DOI:** 10.1038/s41598-023-47382-4

**Published:** 2023-11-16

**Authors:** Omer Lev Ari, Itai Hazan, Jacob Moran-Gilad, Tomer Kerman, Erez Tsumi

**Affiliations:** 1 Department of Ophthalmology, Soroka University Medical Center, Ben-Gurion University of the Negev, Be’er Sheva, Israel; 2 Clinical Research Center, Soroka University Medical Center and Faculty of Health Sciences, Ben-Gurion University of the Negev, Be’er Sheva, Israel; 3 Joyce and Irving Goldman Medical School, Faculty of Health Sciences, Ben-Gurion University of the Negev, Be’er Sheva, Israel; 4 Department of Health Policy and Management, School of Public Health, Faculty of Health Sciences, Ben Gurion University of the Negev, Be’er Sheva, Israel

**Keywords:** Diseases, Eye diseases, Infectious diseases, Epidemiology, Paediatric research

## Abstract

The COVID-19 pandemic notably influenced the transmission of infectious diseases across various age groups. In this study, we assessed its impact on pediatric acute conjunctivitis trends in southern Israel. We analyzed acute conjunctivitis diagnoses from 2017 to 2022, categorizing them into pre-lockdown, lockdown, and post-lockdown intervals. A control group of non-infectious dermatologic conditions was included. Time-series analysis, adjusted for seasonality, was employed. Pre-lockdown data indicated steady conjunctivitis diagnoses, primarily in winter. Post-lockdown interval exhibited an added summer peak before the regular winter surge. The lockdown saw a 56% decline in diagnoses, most pronounced in younger ages. Post-lockdown observed a 7% overall drop with age-specific variations. The acute conjunctivitis IRR was 0.44 (95% CI 0.39–0.49) during lockdowns and 0.93 (95% CI 0.86–1.02) post-lockdowns. Control group IRRs were 0.84 (95% CI 0.78–0.89) and 0.90 (95% CI 0.84–0.96), respectively, with the 0–5 age range demonstrating significant disparities. Pediatric acute conjunctivitis in southern Israel decreased significantly during the pandemic. Post-lockdown patterns varied by age group. An unusual summer peak in cases was observed post-lockdown; this peak may be influenced by a combination of altered behaviors in the summer and possibly increased susceptibility to infection.

## Introduction

The COVID-19 pandemic began in December 2019 and spread worldwide, transmitting via respiratory droplets, causing severe illness and subsequent deaths. The pandemic was officially declared by the world health organization (WHO) in March 2020. The lack of vaccines and suitable treatment left social distancing as one of the only tools to control disease transmission^1^. In addition to the decline in positive Coronavirus cases, social distancing influenced other pathogen transmissions with a decrease in other common viral and non-viral infections, including non-respiratory infectious diseases among children^2–4^.

Conjunctivitis, one of the most common pediatric infectious diseases worldwide, is accountable for up to 2% of United States (US) primary care visits. Infectious conjunctivitis, viral or bacterial, has been known to facilitate outbreaks in a seasonal pattern, highly transmittable during the winter^5–7^ and spread via close personal contact, crowding, or through exposure to droplets found in the air^8^. Conjunctivitis presentation can vary in severity, and its highly contagious forms have been documented to compel the closure of various academic and health facilities, bearing substantial costs on society^9^.

Previous studies regarding the impact of the COVID-19 pandemic on ocular infections have shown lower rates of conjunctivitis during the COVID-19 lockdown in Spain, Turkey, and the US^7, 10, 11^. In this study, we aim to evaluate the impact of the COVID-19 pandemic on pediatric ophthalmic infection visits in clalit health ambulatory services in the southern district during and after the pandemic’s restrictions. The study hypothesis is that there are differences in the acute conjunctivitis diagnoses trends among the pediatric population throughout the COVID-19 pandemic.

## Materials and methods

### COVID-19 lockdowns

Coronavirus first official case in Israel appeared on February 27, 2020. In an attempt to manage and reduce the national spread of the virus infections, the Israeli government implemented social distancing measures, such as mandatory mask wear, limitations on the number of people allowed to congregate in closed spaces, a two-meter distance between people in public areas and mandated three periods of general lockdown to diminish COVID-19 virus transmission from March 2020 to February 2021. The lockdowns include the closure of kindergartens, schools, restaurants, workplaces, and other sectors. Each lockdown has been gradually lifted following a decline in positive cases^12^. Israel’s first lockdown occurred from March 25, 2020, to May 3, 2020, followed by a second lockdown from September 18, 2020, to October 18, 2020; the third and final lockdown was from December 27, 2020, to February 2021^13,14^.

### Study design

This retrospective study was conducted on the Clalit health services database, covering the medical records of patients diagnosed with acute conjunctivitis in Clalit ambulatory services in Israel’s southern district between March 2017 to February 2022. Conjunctivitis diagnosis was defined as a visit in which acute conjunctivitis was coded with the following international code of disease (ICD)-9: 372.0X, excluding acute atopic conjunctivitis 372.05 and acute chemical conjunctivitis 372.06.

To eliminate the potential changes in population behaviors in the post-pandemic period, especially regarding the fear of medical visits for non-severe illnesses, we created a control group consisting of non-infectious dermatologic conditions, which were not supposed to be influenced by COVID-19 activity. These diagnoses were defined as visits in which the following ICD-9 codes were coded during the study intervals: Erythematosquamous dermatosis 690.x, Atopic dermatitis and related conditions 691.x, Contact dermatitis and other eczema 692.x, Psoriasis and similar disorders 696.x and Lichen 697.x.

Each year of our study starts in March and ends in February, dividing the years into intervals corresponding with the pandemic timelines. The patients were divided into three parallel intervals – before, during, and after COVID-19 lockdowns. The first– pre-lockdowns interval includes patients diagnosed with acute conjunctivitis before the pandemic was introduced and covers three consecutive years (March 2017–February 2020), serving as a baseline. The second – lockdowns interval included patients diagnosed with acute conjunctivitis during the pandemic and lockdowns (March 2020–February 2021), and the third – post lockdowns interval consisted of patients diagnosed following the final lockdown, covering the era in which COVID restriction gradually lifted (March 2021–February 2022). We chose to examine three consecutive years for the pre-lockdown interval to demonstrate the trend of acute conjunctivitis diagnosis. Every interval was assessed by age groups: 0–12 months, 1–5 years, 6–11 years, and 12–18 years, corresponding with the Israeli educational framework. Seasons were divided according to the Israeli seasons: December, January, and February—winter; March, April, and May—spring; June, July, and August—summer; September, October, and November—fall. The seasonality nature of infection was evaluated through the monthly diagnosis incidence. Additional background characteristics, including sex, socioeconomic status, and ethnicity, as well as data regarding the conjunctivitis episode, such as the consultation date and the diagnosing physician’s medical specialty, were captured.

### Clalit health services database

Clalit health services (CHS) is the most significant health maintenance organization (HMO), insuring and providing medical services to the majority of the Israeli population. In the southern district, about 75% of the pediatric population receives medical services from CHS^13,14^. The database includes all the medical and personal records of patients insured in Clalit’s southern district. CHS information is entirely digitized and constantly fed into a central database. Data was extracted from CHS using Clalit’s data sharing platform powered by MDClone (https://www.mdclone.com).

### Statistical analysis

We derived rates of acute conjunctivitis and dermatologic diseases per 100,000 children across each age group and then compared the monthly disease-specific rates for every study year. For modeling the seasonal incidence rate ratios (IRRs), we utilized time-series analysis^15,16^. The adjustment for seasonality was achieved by incorporating harmonic terms (sines and cosines) for annual and semiannual patterns. To examine the pandemic’s impact, we delineated three distinct periods: Pre-lockdowns, Lockdowns, and Post-lockdowns. Estimates from the Lockdown and Post-lockdown periods were contrasted with the expected rates from the Pre-lockdown period using quasi-Poisson regression modeling. The fit of the quasi-Poisson regression model was ascertained through visual assessments of the correlograms (Auto-Correlation and Partial Auto-Correlation Functions) and an analysis of residuals. Tests were two-sided, and results with a *P* value of < 0.05 were considered statistically significant. All statistical procedures were executed using R v.3.6.1 (http://www.R-project.org).

### Ethics approval and Consent to Participate

This cross-sectional, population-based study was approved by the Clalit Health Services Research Ethics Committee and was performed in line with the principles of the Declaration of Helsinki. The study is based on an anonymous registry; therefore, the need for informed consent was waived, the approval was granted by the Soroka Medical Center Institutional Review Board (protocol number: 0199-22-SOR). The study is based on an anonymous registry; therefore, the need for informed consent was waived.

## Results

During the study, 135,759 pediatric patients were diagnosed with acute conjunctivitis between March 2017 and February 2022. As shown in Table 1, the final analysis consists of 91,005 patients in the pre-lockdowns intervals (N_1_ = 31,855, N_2_ = 29,837, N_3_ = 29,313), 14,093 patients during the lockdowns interval, and 30,661 patients in the post-lockdowns interval. Overall, the mean age for diagnosis was 4.4 years (SD ± 4.6). Among all study groups, males were more likely to be diagnosed with acute conjunctivitis (*p* < 0.001). The majority were from a medium socioeconomic status (*p* < 0.001), and the most prevalent ethnicity was Jewish. Overall, 57% of the diagnoses were made by pediatricians, 26% by general practitioners, 9.5% by other medical specialties, and 6.7% by ophthalmologists.

**Table 1:** Demographics of Pediatric Conjunctivitis Episodes, According to Study Intervals.

Characteristic	Pre-lockdowns^b^ interval 1 N = 31,855	Pre-lockdowns^c^ interval 2 N = 29,837	Pre-lockdowns^d^ interval 3 N = 29,313	Lockdowns^e^ interval N = 14,093	Post-lockdowns interval^f^ N = 30,661	Overall N = 135,759	*p*-value
Age, years							** < 0.001**
Mean ± SD (N)	4.3 ± 4.6 (31,855)	4.3 ± 4.6 (29,837)	4.4 ± 4.6 (29,313)	5.3 ± 5.0 (14,093)	3.9 ± 4.1 (30,661)	**4.4 ± 4.6 (135,759)**	
Median (IQR)	2.3 (1.0, 6.2)	2.2 (1.0, 6.2)	2.4 (1.1, 6.4)	3.3 (1.2, 8.7)	2.2 (1.1, 5.0)	**2.3** **(1.1, 6.2)**	
Range	0.0, 18.0	0.0, 18.0	0.0, 18.0	0.0, 18.0	0.0, 18.0	**0.0, 18.0**	
Male gender, n (%)	17,281 (54%)	16,452 (55%)	15,926 (54%)	7979 (57%)	16,590 (54%)	**74,228 (55%)**	** < 0.001**
Socioeconomic score, n (%)							** < 0.001**
High	2877 (11%)	2538 (10%)	2218 (8.9%)	1129 (9.4%)	2100 (8.1%)	**10,862 (9.4%)**	
Medium	16,988 (62%)	15,834 (63%)	16,174 (65%)	7565 (63%)	17,160 (66%)	**73,721 (64%)**	
Low	7369 (27%)	6876 (27%)	6621 (26%)	3255 (27%)	6764 (26%)	**30,885 (27%)**	
Ethnicity, n (%)							
Arab	9100 (30%)	8398 (30%)	7741 (28%)	3906 (30%)	8229 (29%)	**37,374 (29%)**	
Jewish	21,240 (70%)	19,805 (70%)	19,879 (72%)	9328 (70%)	20,372 (71%)	**90,624 (71%)**	
Medical Specialties^a^, n (%)							** < 0.001**
General	10,704 (34%)	9624 (32%)	8245 (28%)	3404 (24%)	3404 (24%)	**8080 (26%)**	
Ophthalmology	2073 (6.5%)	1952 (6.5%)	2214 (7.6%)	1764 (13%)	1764 (13%)	**2065 (6.7%)**	
Paediatrics	18,476 (58%)	17,430 (58%)	17,721 (60%)	7799 (55%)	7799 (55%)	**17,575 (57%)**	
Other	580 (1.8%)	809 (2.7%)	1111 (3.8%)	2926 (9.5%)	2926 (9.5%)	**2926 (9.5%)**	

^a^Medical specialties are according to the medical field of expertise. ^b^March 2017–February 2018. ^c^March 2018–February 2019. ^d^March 2019–February 2020. ^e^March 2020–February 2021. ^f^March 2021–February 2022. Significant values are in bold.

As described in Table 2, there was a decrease in acute conjunctivitis diagnoses in all age groups during the lockdowns interval compared to the mean pre-lockdowns diagnosis rates, a reduction of 56% overall, which was more prevalent among the younger age groups; a 61% drop in the 0–1 age group, 62% drop in the 1–5 age group, 40% in the 6–11 age group, and 39% in the 12–18 age group during the pandemic lockdowns. There was an overall 7% decrease in the incidence of acute conjunctivitis diagnoses in the post-lockdowns interval as compared to the pre-lockdowns interval, although not all were affected similarly; a 5% decline among the 0–1 age group, 20% decline in the 6–11 age group, and 31% decline in the 12–18 age group. An increase of 4% was demonstrated among the 1–5 age group.

**Table 2:** Yearly Incidence Rate/100,000 Children – Stratified by Age.

Age	Mean pre-lockdowns^a^ interval—cases/ 100,000 children	Lockdowns^b^ interval		Post-lockdowns^c^ interval		
		Number of cases/ 100,000 children	% Change (From Mean Pre-lockdowns interval	Number of cases / 100,000 children	% Change (From Lockdowns interval)	% Change (From Mean Pre-lockdowns interval)
0–1	45,350	17,814	61% decrease	43,079	142% increase	5% decrease
1–5	19,312	7371	62% decrease	20,078	172% increase	4% increase
6–11	6011	3623	40% decrease	4827	33% increase	20% decrease
12–18	4554	2757	39% decrease	3128	13% increase	31% decrease
Overall	12,542	5517	56% decrease	11,701	112% increase	7% decrease

^a^March 2017–February 2020. ^b^March 2020–February 2021. ^c^March 2021–February 2022.

**Figure 1:**
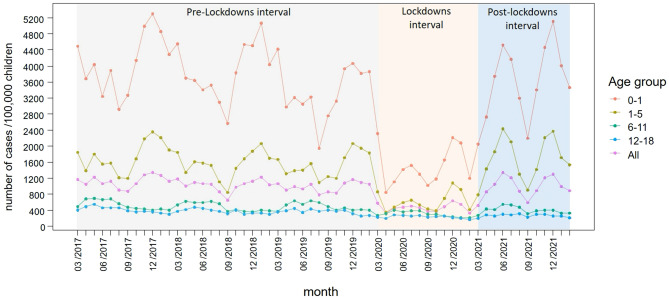
Conjunctivitis Episodes Fluctuations Timeline.

Infectious conjunctivitis diagnoses fluctuations among all ages were stable in the pre-COVID-19 pandemic years (2017–2020), as shown in Fig. 1. There was a higher incidence of cases occurring during the winter (November–February), with fewer episodes of acute conjunctivitis among the pediatric population during the summer (June–September). As described above, between March 2020 and March 2021, during the pandemic lockdowns, the rates of acute conjunctivitis were lower, followed by elevated rates in the post-lockdown months of 2021. There was an additional peak of conjunctivitis diagnosis in post-lockdown times, which occurred during the end of the spring, lasting through the summer, followed by a typical winter seasonal peak.

The time-series analysis (Table 3), showed variations in the IRR of acute conjunctivitis among different age groups during the lockdowns interval, in contrast to the control group. For all age groups, the acute conjunctivitis group experienced a significant decrease, resulting in an IRR of 0.44 (95% CI 0.39–0.49), compared to the control group’s IRR of 0.84 (95% CI 0.78–0.89). In the 0–1 years age group, acute conjunctivitis had an IRR of 0.39 (95% CI 0.34–0.46) while the control group had an IRR of 0.79 (95% CI 0.73–0.85). Children in the age group of 1–5 showed an IRR of 0.38 (95% CI 0.32–0.45) for acute conjunctivitis, which is a more considerable decline than the control group’s IRR of 0.80 (95% CI 0.75–0.86). For the 6–11 age group, acute conjunctivitis had an IRR of 0.60 (95% CI 0.55–0.66), while the control group indicated an IRR of 0.89 (95% CI 0.83–0.96). Remarkably, adolescents aged 12–18 experienced a decrease in IRR for acute conjunctivitis, at 0.61 (95% CI 0.56–0.66), whereas the IRR of the control group remained relatively consistent at 0.97 (95% CI 0.90–1.04).

**Table 3:** Incidence of Acute conjunctivitis by different time periods and a negative control sensitivity analysis. Results of time series analysis^b^.

Study group	Period	All ages		0–1 years		1–5 years		6–11 years		12–18 years	
		IRR (95% CI)^a^	*p*-value	IRR (95% CI)^a^	*p*-value	IRR (95% CI)^a^	*p*-value	IRR (95% CI)^a^	*p*-value	IRR (95% CI)^a^	*p*-value
Acute conjunctivitis group	Pre-lockdowns^d^	–		–		–		–		–	
	Lockdowns^e^	0.44 (39, 0.49)	< 0.001	0.39 (0.34, 0.46)	< 0.001	0.38 (0.32, 0.45)	< 0.001	0.60 (0.55, 0.66)	< 0.001	0.61 (0.56, 0.66)	< 0.001
	Post-lockdowns^f^	0.93 (0.86, 1.02)	0.13	0.95 (0.85, 1.06)	0.4	1.04 (0.93, 1.16)	0.5	0.80 (0.75, 0.87)	< 0.001	0.69 (0.63, 0.74)	< 0.001
Control group^c^	Pre-lockdowns^d^	–		–		–		–		–	
	Lockdowns^e^	0.84 (0.78, 0.89)	< 0.001	0.79 (0.73, 0.85)	< 0.001	0.80 (0.75, 0.86)	< 0.001	0.89 (0.83, 0.96)	0.005	0.97 (0.90, 1.04)	0.4
	Post-lockdowns^f^	0.90 (0.84, 0.96)	0.001	0.91 (0.85, 0.98)	0.021	0.87 (0.81, 0.93)	< 0.001	0.94 (0.87, 1.01)	0.093	0.99 (0.92, 1.06)	0.7

^a^*IRR* Incidence Rate Ratio, *CI* Confidence Interval, ^b^Adjusted for seasonality. ^c^non-infectious skin conditions group. ^d^March 2017–February 2020. ^e^March 2020–February 2021. ^f^March 2021–February 2022.

During the post-lockdowns interval (Table 3), the acute conjunctivitis group showed an overall IRR of 0.93 (95% CI 0.86–1.02) as compared to an IRR of 0.90 (95% CI 0.84–0.96). For the 0–1 age bracket, the acute conjunctivitis group indicated an IRR of 0.95 (95% CI 0.85–1.06), in contrast to the control group’s IRR of 0.91 (95% CI 0.85–0.98). Children aged 1–5 had an IRR of 1.04 (95% CI 0.93–1.16) for acute conjunctivitis, which is a higher value than the control group’s IRR of 0.87 (95% CI 0.81–0.93). In the 6–11 age group, the IRR for acute conjunctivitis was 0.80 (95% CI 0.75–0.87), while the control group reported an IRR of 0.94 (95% CI 0.87–1.01). Adolescents between 12 and 18 had an acute conjunctivitis IRR of 0.69 (95% CI 0.63–0.74), and the control group’s IRR stood at 0.99 (95% CI 0.92–1.06).

## Discussion

In this analysis of 135,759 pediatric patients diagnosed with acute conjunctivitis between March 2017 to February 2022, we examined pediatric conjunctivitis infection rates before, during, and after the COVID-19 pandemic restriction and assessed the impact of the pandemic and lockdowns on acute infectious conjunctivitis incidence among the pediatric population in southern Israel CHS. To the best of our knowledge, this is the first study to examine the trends of acute infectious conjunctivitis between three pre-COVID-19 pandemic years and the two pandemic years.

Our study demonstrated a stable trend in the incidence of acute conjunctivitis episodes before the COVID-19 pandemic. During the three COVID-19 lockdowns, we observed a shift in normalcy. The number of diagnosed patients decreased among all ages. This decline was statistically significant in a time series approach, even after adjusting for seasonality. When compared to a control group consisting of non-infectious skin diseases, the decline was notably greater in cases of acute conjunctivitis, suggesting the evident declines represents an actual reduction in the incidence of infection, rather than a change in health seeking behaviors or access to healthcare during this period. The pattern of reduced infection rates among the pediatric population during the COVID-19 pandemic lockdowns was described in other communicable diseases^2,4,17–20^.

The decline in acute conjunctivitis rates can be attributed to the social and physical distancing mandated by governmental restrictions and lockdowns. The public was required to wear a face mask and stay within a limited home range, 100 m limit range on the first lockdown, 500 m on the second, and 1000 m on the third. All regular educational frameworks were closed, and classes were conducted online during the full lockdowns. In the intervals between full lockdowns, attending school grounds and classrooms was permitted for specific school grades, primarily for children in early school grades, depending on the number of positive COVID-19 cases in the resident district. When school attendance met the requirements, classes were performed in small and invariable teaching capsules and carried out on different timeframes. Youth groups and other afterschool events were canceled during and between the lockdowns^21–23^.

The highly contagious syndrome of infectious conjunctivitis has accounted for many outbreaks in various leisure, healthcare, and educational frameworks. Among the pediatric population, the most common transmission is thought to be through direct contact^24^. Poor hand hygiene and sharing contaminated objects can also be attributed to the transmission of infectious conjunctivitis^8^. Hand and personal hygiene campaigns issued by the government, raising awareness for COVID-19 virus transmission, were spread vastly across the country, contributing to lower rates of infectious conjunctivitis. Meticulous hand hygiene practice’s ability to reduce transmission of other common pathogens among the pediatric population was described before^25^.

Lockdown end had different outcomes depending on the age group. For the 6–11 years and 12–18 years age groups, the restriction termination resulted in lower conjunctivitis rates than before the pandemic. The decline in conjunctivitis rates among these age groups was statistically significant in a time series analysis, even after adjusting for seasonal variations. The establishment of better hand hygiene practices mentioned earlier might explain the lower rates of conjunctivitis among these groups, even after lifting COVID-19 restrictions. Despite the positive effect hand hygiene might have, another plausible explanation for reduced post-lockdown conjunctivitis rates is rooted in the mental and behavioral effects of the COVID-19 pandemic. Children and teens suffer higher levels of depression and anxiety, influencing their integration capabilities and social skills, resulting in their gravitation toward online social interactions over real-life gatherings^26^.

Among the younger age groups, 0–1 and 1–5, the rates in the post-lockdowns months had begun to return to their pre-pandemic rates. With a slightly non statistically significant increased incidence among the 1–5 years age group and a subtle decrease among the 0–1 age group. Since conjunctivitis rates before the COVID-19 pandemic reflect regular everyday activity, the pattern seen among these age groups correlates with the return to the usual routine. This might also be clarified by the immaturity of children at these ages, affecting their abilities to perform hygiene practices and to maintain social awareness of pathogen transmission.

Adenovirus, responsible for most viral conjunctivitis, has been known to facilitate outbreaks in a seasonal pattern, typically contagious in the winter and spring^5,7^. Interestingly, we found that during post-lockdown months, there was an additional, non-typical peak, which occurred during the end of the spring, lasting through the summer, similar in form to the winter peak, followed by a usual winter seasonal peak. The pattern of “seasonal switch” was described in a similar study conducted in Israel^14^. Respiratory syncytial virus (RSV) demonstrated a similar non-typical seasonal pattern in Australia and Israel with unparalleled virus outbreaks during the summer occurring during the pandemic restrictions gradually lifting^27,28^. The added peak lasting through the summer supports human behavior as the etiology of the seasonality pattern of acute viral conjunctivitis. The fact that individuals spend more time indoors in proximity to others might play a more significant role in the higher incidence of acute conjunctivitis during the winter than the low temperatures considered favorable to some pathogens.

The additional summer peak could also be a non-infectious form of acute conjunctivitis. The lack of exposure to common allergens among children at a young age due to COVID-19 pandemic restrictions, along with over-cleanliness (i.e., Hygiene hypothesis), resulted in higher rates of allergic manifestations of conjunctivitis^29^. A previous study examined the differential diagnosis and treatment for patients presenting with “pink eye” to general practitioners and ophthalmologists in nine countries; demonstrated a higher tendency for diagnoses of allergic conjunctivitis among general medical practitioners compared to an ophthalmologist during the spring and summer seasons^30^. In our study, a large portion of the diagnoses was made by pediatricians and general practitioners, therefore strengthening our results regarding the lower rates of infectious conjunctivitis. Pediatricians tend to diagnose patients with pink eye as “allergic conjunctivitis” during the spring and summer; the fact that those doctors, who made the majority of the diagnoses gave “conjunctivitis” diagnoses – strengthens our results.

The concept of “immunity debt” may also underpin the atypical surge in acute conjunctivitis cases post-lockdown. The prolonged isolation during lockdown likely diminished exposure to various pathogens, leading to an accumulation of ‘immunity debt’. Once social interactions resumed post-lockdown, heightened susceptibility due to this ‘debt’ could have contributed to the uncharacteristic summer peak in conjunctivitis cases, as suggested^31,32^. While this concept may hold true for infectious agents transmitted via the respiratory tract (e.g. meningococcal illness or influenza), its applicability to pathogens transmitted via direct contact such as many causative organisms of conjunctivitis remains questionable and deserves further study.

## Conclusions

Acute conjunctivitis episodes among the southern Israel pediatric population have dropped significantly during the COVID-19 pandemic and lockdowns among all ages. With restrictions termination, there was an elevated rate of conjunctivitis diagnoses during the summer, followed by a typical winter rate, which might suggest that the seasonality nature of infectious conjunctivitis might be associated to a greater extent with human behavior during the winter than with the temperature itself. The lockdowns have changed the behavior of teenagers, who also, after the lockdowns, kept lower rates of this disease. Future studies are needed to evaluate the long-lasting effects of the COVID-19 pandemic on acute conjunctivitis among the pediatric population.

## Limitations

This study is based on the assumption that the absence of diagnoses is associated with the absence of morbidity. Theoretically, the lack of diagnoses in the system may be due to patients opting to avoid medical consultation during the pandemic due to fear or reduced access to health care, which can occur in diseases perceived as “mild” by the public, such as conjunctivitis. Lavista et al. evaluated the correlation between the COVID-19 pandemic restrictions and internet search interest regarding infectious conjunctivitis; has demonstrated a vast decline in the rates of viral conjunctivitis in a large-scale population study using online search metrics combined with data on reduced emergency department-related visits^7^. Pediatricians account for the majority of diagnoses in our study. Although acute infectious conjunctivitis is one of the most common pediatric infections and accounts for many encounters and in-office consultations among general practitioners and pediatricians^33,34^, some might claim that the diagnoses might not be accurate. While other eye diseases might be confused with infectious conjunctivitis due to overlapping manifestations, studies on antibiotic prescribing habits among non-ophthalmologists found that the main difficulty is differentiating viral from bacterial etiology and not determining whether it is infectious^35,36^. Our study did not distinguish viral from bacterial etiology, and we did not include etiological data.

## Data Availability

The data generated and analyzed during the current study are not publicly available due to hospitalization privacy but are available from the corresponding author upon reasonable request.

## Abbreviations

WHO: World Health Organization
US: United States
CHS: Clalit Health Services database
ICD-9: International Code of Disease
HMO: Health Maintenance Organization
IQR: Interquartile range
SD: Standard deviation
